# Functional Selectivity of Dopamine D_1_ Receptor Signaling: Retrospect and Prospect

**DOI:** 10.3390/ijms222111914

**Published:** 2021-11-03

**Authors:** Yang Yang

**Affiliations:** Department of Pharmacology, Penn State University College of Medicine, Hershey, PA 17033, USA; yangyang@psu.edu

**Keywords:** functional selectivity, dopamine D_1_ receptor, clinical implications

## Abstract

Research progress on dopamine D_1_ receptors indicates that signaling no longer is limited to G protein-dependent cyclic adenosine monophosphate phosphorylation but also includes G protein-independent β-arrestin-related mitogen-activated protein kinase activation, regulation of ion channels, phospholipase C activation, and possibly more. This review summarizes recent studies revealing the complexity of D_1_ signaling and its clinical implications, and suggests functional selectivity as a promising strategy for drug discovery to magnify the merit of D_1_ signaling. Functional selectivity/biased receptor signaling has become a major research front because of its potential to improve therapeutics through precise targeting. Retrospective pharmacological review indicated that many D_1_ ligands have some degree of mild functional selectivity, and novel compounds with extreme bias at D_1_ signaling were reported recently. Behavioral and neurophysiological studies inspired new methods to investigate functional selectivity and gave insight into the biased signaling of several drugs. Results from recent clinical trials also supported D_1_ functional selectivity signaling as a promising strategy for discovery and development of better therapeutics.

## 1. A Brief Introduction of Functional Selectivity

The term functional selectivity [1] was first introduced in 1994 and soon thereafter other reports referred to this phenomenon alternately as “agonist trafficking of signaling”, “differential engagement”, or “biased agonism”. The concept of functional selectivity essentially means that a ligand may have different actions at two or more signaling pathways mediated by the same receptor. Ligand actions may range from full agonism at one pathway and antagonism at another. Before functional selectivity was introduced, a ligand was defined as either an agonist or antagonist for one canonical signaling pathway mediated by the target receptor. This simplified notion had to be abandoned once it became clear that multiple signaling pathways could be involved by the activation of a receptor. This pharmacological insight contributed significantly to the development of the concept of functional selectivity. Meanwhile, functional selectivity also benefited from the progress on structural biology where the discovery of heterodimers and some detailed crystal structures inspired understanding of additional signaling properties of a receptor. The concept of functional selectivity has evolved. Initially, functional selectivity was studied using a “paired model”, such that a newly discovered receptor signaling pathway was compared with the traditional canonical pathway. Now, the “network model” is more appreciated whereby all signaling pathways related to a receptor are treated equally and the signaling complex as a whole accounts for the pharmacological property of a ligand. Functional selectivity has become a major pharmacological research front and more relevant as additional signaling pathways were discovered. The full signaling properties of many new and some “old” ligands have been studied or reinvestigated to better understand their engagement of targets in more precise ways and potentially to improve their therapeutic index. To grasp the significance of functional selectivity of dopamine D_1_ ligands, it is important first to review all canonical and novel signaling pathways related to dopamine D_1_-like receptors (D_1_Rs).

## 2. G Protein-Dependent Cyclic Adenosine Monophosphate (cAMP) Phosphorylation

**Figure 1:**
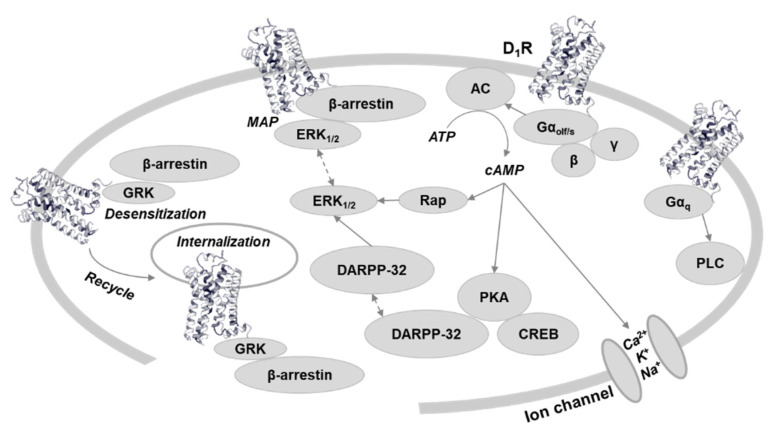
Dopamine D_1_ receptor-related signaling. The traditionally canonical G protein coupled cAMP signaling potentially could be subdivided based on G protein subtype [3] and PKA subunit [4]. G protein independent, β-arrestin-related signaling acts through MAP kinase phosphorylation [5], and has cross talk with cAMP signaling [6,7]. Receptor recycling is also regulated by β-arrestin. Regulation of ion channels could be through cAMP [8]. Gα_q_ dependent PLC signaling is controversial [9]. Abbreviations: D_1_R, dopamine D_1_ receptor; AC5, adenylate cyclase type 5; PKA, protein kinase A; ERK, extracellular-signal-regulated kinase; GRK, G protein-coupled receptor kinase; DARPP-32, Dopamine and cAMP-related phosphoprotein 32KDa; Rap, a small GTPase; CREB, cAMP response element-binding protein; PLC, phospholipase C.

Dopamine receptors belong to the G-protein-coupled receptor (GPCR) group of receptors and classically have been linked to adenylate cyclase. D_1_Rs interact with Gα_s/olf_ to stimulate adenylate cyclase and produce cAMP (Figure 1). Nobel Laureate Paul Greengard and his colleagues made a series of discoveries in the 1970s [2] that revealed that dopamine interacts with receptors to cause an increase in cAMP, activates protein kinase A (PKA), and in turn phosphorylates other proteins. Therefore, G protein coupled adenylate cyclase activation traditionally was used as the canonical signaling pathway for D_1_R.

G proteins are a family of proteins made up of subunits Gα, Gβ, and Gγ. D_1_Rs stimulate adenylate cyclase primarily through Gα_s_. The Gα_s_ family is comprised of Gα_s_ and G_olf_, the latter named for its predominant expression in the olfactory system. Studies on Gα_olf_ knock-out mice suggested that Gα_olf_ may play an essential role in D_1_R-mediated cAMP accumulation [10,11]. Gα_olf_ knock-out mice showed no hyperlocomotor response to the D_1_ agonist SKF81297, and their striatal D_1_Rs had decreased affinity for dopamine. The adenylyl cyclase response to dopamine in the caudate/putamen and nucleus accumbens were also decreased dramatically, but prefrontal cortex signaling remained unaffected. Studies on the striatum of Parkinson’s disease patients also suggested that Gα_olf_ plays the essential role in D_1_R-mediated cAMP [12]. The hallmark of Parkinson’s disease is decreased dopamine in the striatum. Interestingly, Gα_olf_ in this region is less abundant in Parkinson’s patients, whereas Gα_s_ is highly expressed. The combination of dopamine deficiency and Gα_olf_ being sparsely expressed suggested that the coupling of D_1_Rs to adenylate cyclase is mediated primarily by Gα_olf_ or the switch/shift between Gα_s_ and Gα_olf_.

The mRNA of other G proteins are also expressed in the striatum. For example, G protein γ_7_ subunit mRNA was detected within rat brain areas that aligned with striatum-enriched adenylyl cyclase, dopamine receptors, and Gα_olf_. This suggested that γ_7_ may be part of the Gα_olf_-containing complex that couples dopamine receptors selectively to adenylyl cyclase [13]. Another study used a ribozyme strategy to suppress the expression of the G protein γ_7_ subunit in HEK 293 cells stably expressing the human D_1_Rs, and revealed a significant attenuation of D_1_ agonist SKF81297-stimulated adenylyl cyclase activity [14]. This evidence supported the potentially important function of the G protein γ_7_ subunit on D_1_R-mediated cAMP signaling.

Along with improved understanding of the interaction between D_1_Rs and each G protein subunit, there is ongoing progress regarding functional selectivity of G protein subtype-specific signaling. Although there are currently no D_1_ ligands being developed to target specific G protein subunits, such compounds have been reported for the C-C chemokine receptor 5 (CCR5) [3]. Lorenzen et al. studied four chemokine analogs and found that some analogs were super agonists for G_q/11_ activation, whereas other analogs displayed a signaling bias for G_i/o_. Their results demonstrated that ligands can elicit G protein subtype-specific signaling bias and cause receptors to couple preferentially to one subtype of G protein signaling over others. This is inspiring for the pharmacology field. A timely study is needed to investigate this interesting phenomenon since it is reasonable to assume that some dopamine ligands may also engage each G protein subtype differently, providing a more targeted action.

## 3. β-arrestin-related Signaling and Mitogen-Activated Protein (MAP) Kinase Activation

In addition to G protein-dependent adenylyl cyclase signaling, research has demonstrated the importance of G protein independent β-arrestin-related signaling. Originally, arrestin was thought to only promote receptor desensitization and internalization. It blocks GPCR coupling to G proteins, preventing GPCR’s activation (desensitization), or links GPCR to elements of internalization machinery. Starting in the 1970s, Nobel Laureate Robert Lefkowitz and his colleagues made several discoveries related to β-adrenergic receptors, and β-arrestin related signaling is one of their concomitant discoveries. To date, it has been reported widely in several receptor systems, including β-adrenergic [5], opioid [15], cannabinoid [16], angiotensin II type 1 [17], 5-HT2A [18], apelin [19], growth hormone secretagogue [20], sphingosine 1-phosphate [21], and dopamine D_2_-like receptors (D_2_Rs) [22,23]. Regarding D_1_Rs, there are a few reports that suggested the existence of an interaction between D_1_R and β-arrestin and indicated the involvement of MAP kinase phosphorylation (Figure 1), but many of them did not specify or propose D_1_R-mediated β-arrestin signaling.

Chen et al. used co-immunoprecipitation to show that extracellular signal-regulated kinases 1 and 2 (ERK_1/2_) formed stable heterotrimeric complexes with the D_1_R and β-arrestin2. In cells transfected with the dominant negative mutant of β-arrestin2, however, the formation of such complexes was inhibited substantially [24]. Similarly, Urs et al. utilized a D_1_R knockout mouse model and demonstrated that formation of the β-arrestin2 and ERK_1/2_ complexes was also blunted [25,26]. In contrast, others stimulated striatal or prefrontal cortex D_1_Rs in vivo or in vitro with the D_1_ selective agonist SKF38393 and showed ERK_1/2_ was phosphorylated [24,27,28]. These studies provided the initial evidence of D_1_R-mediated β-arrestin signaling and its function through ERK_1/2_. In addition to ERK_1/2_, several studies indicated the involvement of other MAP kinases. Zhen et al. showed SKF38393 increased activation of p38 MAP kinase and c-Jun amino-terminal kinase in SK-N-MC neuroblastoma cells that endogenously express D_1_Rs, whereas ERK activity was not affected [29]. This study suggested that D_1_R-related MAP kinase phosphorylation could be cell-type specific.

Another interesting finding is that D_1_R-related MAP kinase phosphorylation may also be dependent of PKA. Studies in parkinsonian mouse models indicated that a PKA substrate, dopamine and cAMP-regulated 32 kDa phosphoprotein (DARPP-32), was critically involved in D_1_R-mediated ERK_1/2_ phosphorylation. A mutation on the phosphorylation site of DARPP-32 reduced activation of ERK, whereas sensitization of DARPP-32 led to increased activation of ERK_1/2_ [6,30]. The selective PKA inhibitor Rp-cAMP eliminated the activations of ERK_1/2_, p38 MAP kinase, and c-Jun amino-terminal kinase [27,29]. In contrast, cAMP directly activated a guanine-nucleotide-exchange factor that stimulates Rap GTPase and promotes the MAP kinase cascade [7,31]. These studies suggested there may be potential cross talk between D_1_R-mediated cAMP and β-arrestin-related signaling.

## 4. Regulation of Ca^2+^, K^+^, and Na^+^ Channels

Many studies have indicated D_1_R involvement in the regulation of ion channels. Voltage-dependent Ca^2+^ channels (L-, N-, and P/Q-type) play critical roles in balancing intracellular Ca^2+^ concentrations that are key for neurotransmitter release and synaptic plasticity [32]. Using whole-cell voltage-clamp techniques, Surmeier et al. showed that the application of D_1_ agonists reduced N- and P-type Ca^2+^ currents, but enhanced L-type currents. The differential regulation of Ca^2+^ currents by D_1_ agonists helps to explain the diversity of effects that D_1_Rs have on synaptic integration and plasticity [33]. Hernandez-Lopez et al. furthered the study of D_1_R effects on L-type Ca^2+^ current. They examined the impact of D_1_ agonists at depolarized and hyperpolarized membrane potentials and showed that D_1_R activation either can inhibit or enhance evoked activity, depending on the level of membrane depolarization. Interestingly, the effects on evoked activity at membrane potentials were blocked by the L-type Ca^2+^ channel antagonists nicardipine or calciseptine, and were occluded by the agonist BayK8644. These data indicated that the D_1_R-mediated effects occurred through the L-type Ca^2+^ channel [34]. For N-type Ca^2+^ channels, coimmunoprecipitation showed the existence of a D_1_R-N-type Ca^2+^ channel signaling complex in the prefrontal cortex. This complex had a direct receptor-channel interaction. D_1_ agonists not only regulated N-type Ca^2+^ channel distribution but also inhibited influx Ca^2+^ current. Consequently, neuronal transduction was attenuated [35,36]. The cAMP/PKA/DARPP-32 signaling cascade appeared to mediate these effects on Ca^2+^ channels, as cyclic AMP analogs mimicked the effects of D_1_ agonists [33].

The D_1_ agonist SKF81297 or SKF82957 in combination with the D_2_ agonist quinpirole increased spike firing of nucleus accumbens neurons via inhibition of a slow A-type K^+^ current. This enhancement was prevented by inhibitors of PKA or G_βγ_ and enabled by intracellular perfusion with G_βγ_. These data suggested that the underlying mechanism of D_1_R and D_2_R cooperativity in mediating the slow A-type K^+^ current was by activation of specific subtypes of adenylyl cyclases released by G_βγ_ from the G_i/o_-linked D_2_R in combination with Gα_s_-linked D_1_R [37]. The D_1_ agonists SKF81297 or dihydrexidine induced prolonged membrane depolarization and excitability of fast-spiking interneurons in the prefrontal cortex. Voltage-clamp analyses revealed that this mimicked dopamine-suppressed inward rectifying K^+^ current and can be reduced by the D_1_ antagonist SCH23390 [38,39,40,41]. Although the precise mechanism underlying D_1_R-mediated K^+^ current changes has not been fully understood, studies have suggested possible options: the direct interaction of cAMP with K^+^ channels and the involvement of D_1_R-mediated cAMP/PKA signaling. The first reason is that the effect of D_1_R stimulation on K^+^ current can be mimicked by the adenylyl cyclase activator forskolin and the active cAMP analog Sp-cAMP. The second reason is that the inhibition of PKA with either PKI, Rp-cAMP, or the protein phosphatase inhibitor okadaic acid abolished D_1_R modulation [40,41].

D_1_Rs also appear to impact Na^+^ channels. For example, the D_1_ agonist SKF38393 reduced the peak Na^+^ current amplitude in rat striatal neurons and subsequently depressed striatal neuron excitability. These effects were reversed by the D_1_ antagonist SCH23390 [42,43]. Intracellular loading of PKA mimicked D_1_R-mediated Na^+^ current inhibition, and diffusion of the PKA inhibitor PKI into the cytosol of neurons blocked it, suggesting the involvement of PKA [44]. Schiffmann et al. suggested the critical role of phosphorylated DARPP-32 as part of this pathway since its injection reduced the Na^+^ current amplitude [45]. This line of evidence suggested that D_1_R regulation of ion channels may be a subsequent event of D_1_-mediated G protein-dependent cAMP signaling. On the other hand, Cantrell et al. reported that phosphorylation of Ser573 on the Na^+^ channel α subunit was critical for D_1_R-mediated effects on the Na^+^ current since this site was phosphorylated by D_1_R activation [46]. Since the structure of the ion channel itself potentially could play a critical role, as shown by the Ser 573 study, it is also reasonable to assume that there is a more “direct” interaction between the D_1_R and ion channel. A timely study is needed to investigate this interesting hypothesis. More importantly, if some dopamine ligands can engage each ion channel differently, they may provide a more targeted action and potentially lead to better therapeutic implications.

## 5. Phospholipase C (PLC) Activation

D_1_R-mediated PLC signaling was once proposed as a novel target, but controversies occurred, and it now is considered to be purported. The possibility that D_1_Rs may function through PLC first was reported in a series of studies on adenylate cyclase type 5 (AC5), a dopamine sensitive-adenylate cyclase. Genetic disruption of the AC5 isoform led to loss of adenylyl cyclase activity after administering the D_1_ agonist SKF38393, and this was accompanied by a decrease in the expression of Gα_s_. AC5 null mice also showed parkinsonian-like motor dysfunction. Interestingly, administration of the partial D_1_ agonist SKF38393 improved some of the symptoms, suggesting compensation of D_1_ signaling outside the Gα_s_ mechanism and beyond adenylate cyclase [47,48]. Gα_q_-mediated PLC activation and subsequent Ca^2+^ elevation as a non-cyclase signaling for D_1_Rs was then proposed to explain D_1_R-mediated motor behaviors of the null mice. SKF38393 increased Gα_q_ protein binding to the D_1_R in the striatum, suggesting the possible role of the Gα_q_ protein in D_1_R-mediated PLC activation [49]. Several studies indicated that SKF38393 activated PLC in brain slices, and this action was inhibited selectively by the D_1_ antagonist SCH23390. In addition, dopamine-induced inward Ca^2+^ current was mimicked by the administration of SKF38393 and blocked by SCH23390 [50,51,52].

The most supportive evidence for D_1_R-mediated PLC signaling came from studies using the D_1_ ligand SKF83959 that has small effects on adenylate cyclase but strong efficacy for PLC activation. Interestingly, it induced contralateral rotations in the unilateral 6-OHDA-lesioned parkinsonian rat model, and the rotations were completely blocked by the D_1_ antagonist SCH23390 [53,54]. The involvement of the D_1_R in PLC signaling, however, is still controversial [9] because the behavioral effects of SKF83959 can be explained by several other mechanisms. First, SKF83959 is still a typical partial agonist for adenylate cyclase [55]. Second, non-specific effects on other receptors also could explain the behavioral effects of SKF83959 since it can bind to several GPCRs in micromolar concentrations [56]. Third, it is postulated that D_1_Rs and D_2_Rs form a D_1_/D_2_ heterodimer. Heterodimers have been shown to play a role in functional selectivity in several other GPCR systems [57,58], including the D2/neurotensin NTS1 receptor complex [59] and the D2/trace amine-associated receptor 1 heterodimer [60]. It is very tantalizing to think that the D_1_/D_2_ heterodimer led to PLC signaling. D_1_Rs and D_2_Rs, however, are seldom co-expressed in striatal neurons [61,62,63,64,65], suggesting that the heterodimer mechanism is likely not a major contributor. Collectively, the evidence suggests that D_1_Rs actually may be independent of PLC activation.

## 6. Insight on Functional Selectivity through the Implications of D_1_ Signaling

Dopamine receptors are highly expressed in the brain. The densest area is forebrain where the major dopaminergic terminal fields occur including caudate-putamen and nucleus accumbens. The midbrain (i.e., substantia nigra and ventral tegmental area) also has a high density of dopamine receptors. Olfactory, limbic, and brainstem areas have moderate densities of dopamine receptors. The cerebral cortex has a relatively light density of dopamine receptors but those that are there have important functional implications. In general, the density of D_1_Rs (including D_1_ and D_5_) is higher than that of D_2_Rs (D_2_, D_3_ and D_4_), especially in the cortex where there is a significantly higher overall density and different laminar patterning of D_1_R compared to D_2_R. The D_1_R is preferentially distributed in deeper cortex layers and is proportionally more widespread and expressed within local GABAergic interneuron populations. In the basal ganglia where the density of dopamine receptors is the highest, the segregation of D_1_R and D_2_R is more distinct, with <10% overlap. The GABAergic medium spiny projection neurons of the striatum express D_1_R in the direct pathway and D_2_R in the indirect pathway. These two parallel and segregated pathways form the outflow of the basal ganglia to regulate thalamocortical circuitry [66]. Dysfunction of dopamine receptors in these brain areas play causal roles in many neurological disorders. Therefore, targeting D_1_Rs for therapeutic intervention is attractive. In this section, we try to differentiate D_1_ signaling by highlighting some reports that in retrospect have contributed to the understanding of functional selectivity.

There has been a long history between D_1_R-mediated cAMP and Parkinson’s disease. AC5 is highly concentrated in the striatum. Genetic ablation of the AC5 gene eliminated adenylate cyclase activity stimulated by D_1_ agonists in the striatum, and induced parkinsonian-like motor dysfunction. These findings supported the involvement of D_1_R-mediated AC5 activation in the motor symptoms of Parkinson’s disease [47,48,67]. AC5-produced striatal cAMP binds to the regulatory subunits of PKA that then phosphorylates various proteins such as DARPP-32 and cAMP response element-binding protein (CREB). Although how this signaling leads to D_1_-mediated behavioral effects is still unclear, these downstream molecules are involved in the regulation of gene expression [4]. These lines of evidence encouraged the development of functionally selective dopamine ligands whose cAMP signaling can be biased to the PKA subunit to provide a more targeted action improved therapeutic index.

Recent studies on D_1_R-mediated β-arrestin have yielded several impressive clinical implications. Urs et al. reported that the D_1_R-dependent, β-arrestin-related ERK signal cascade affected morphine-induced psychomotor activation but not reward [24,25], suggesting a separation of therapeutics (e.g., analgesic) from side effects (e.g., addiction). By analyzing transcriptional signatures in humans and mice, Labonte et al. reported that D_1_R-mediated β-arrestin signaling through ERK may effect sex-specific depression [68]. Several studies on rats or mice with mutations that eliminate β-arrestin recruitment showed less locomotor activity [69], more dyskinesia-like behavior [26], enhanced adiposity [20], and impaired memory reconsolidation [70]. Recently, our team used a pair of D_1_ agonists with distinct signaling profiles at β-arrestin recruitment to evaluate rodent behavior in a working memory-related T-maze task [71]. We showed subtle but significant behavioral variation associated with the level of β-arrestin recruitment, suggesting a promising implication of β-arrestin-selective D_1_ agonists on cognitive improvement. Interestingly, there were also clinical implications reported from ligands that bias against β-arrestin. Jiang et al. and other groups reported that β-arrestin activation was related to β-amyloid-induced cognitive impairment [72,73,74]. Since β-amyloid is one of the key players in Alzheimer’s disease, this finding implies that bias against β-arrestin could be a target for limiting β-amyloid-induced cognitive impairment. D_1_ agonists that have less β-arrestin activity may be novel therapeutics. Functionally selective D_1_ agonists that have less β-arrestin activity may have less side effects in Parkinson’s disease because ERK_1/2_ activation correlated with levodopa-induced dyskinesia, whereas blocking ERK_1/2_ activation significantly decreased it [30]. Indeed, recent phase III Parkinson’s disease and phase IIa schizophrenia trials using novel D_1_ agonists with almost no β-arrestin activity have shown highly positive clinical indications [75,76,77,78,79,80].

It is encouraging to see D_1_R signaling has several clinical implications, even though some results seemed to contradict each other. In fact, the concept of functional selectivity was born in part to deal with the fact that different types of bias at a signaling pathway lead to benefits or disadvantages depending on different conditions. Essentially, functional selectivity was founded on the idea of precise targeting. In other words, the bias at one receptor’s whole signaling complex could be adjusted based on different applications to magnify merit.

## 7. Progress on the Structural Biology of Dopamine Receptors

Progress on the structural biology of dopamine receptors has a large impact on the theory of functional selectivity and the discovery of subtype selective ligands, although it is rather slow for D_1_Rs. The crystal structure of D_1_R complexed with a G protein and a non-catechol agonist was reported only recently [81], although predictions of the 3D structure of human D_2_Rs, the binding site, and binding affinities for agonists and antagonists have been around since the 2000s [82]. Over the intervening years, several crystal structures of ligand-bound D_2_R, D_3_R, D_4_R, and D_2_R-G_i_ complexes were reported [83,84,85]. By studying selected transmembrane-5 serine mutations, Fowler et al. showed that receptor conformations were involved in D_2L_R functional selectivity [86]. Using docking simulations and site-directed mutagenesis, Zhang et al. not only reported the crystal structure of the human angiotensin II type 1 receptor in complex with one of its inverse agonists but also identified specific interactions between the angiotensin II type 1 receptor and different ligands. This provided support for the structural basis of ligand recognition and functional selectivity [87].

The heterodimer theory was also enhanced by structural biology progress. Crystal structures of receptor–ligand complexes allow for rational design of novel molecular probes and drugs. Following this strategy, bivalent ligands were synthesized and investigated for the D_2_R/neurotensin NTS1 receptor heterodimer. The results indicated a strong, predominantly NTS1 receptor-mediated β-arrestin-2 recruitment in D_2_R/NTS1 receptor-co-expressing cells [59]. There have been several other studies on the functional selectivity of GPCR heterodimerization [57,58] but because of the sparse co-expression of D_1_Rs and D_2_Rs in the same neuron [61,62,63,64,65], it still is unclear whether the D_1_/D_2_ heterodimer plays a critical role in cell signaling or overt behaviors.

## 8. Potential of Receptor Localization-Related Functional Selectivity

It is commonly known that dopamine receptors function differently in distinctive brain areas. Although there is not yet many focused studies to specifically investigate this, the possibility is high that functional selectivity is involved as a part of the underlying mechanism. More intriguingly, recent studies have shown that besides the primary effects in the central nervous system, dopamine also acts in the pancreas as a peripheral regulator of metabolism, and this action is functionally biased at D_2_Rs [88]. This suggests that the receptor localization-related functional selectivity may be a widespread phenomenon. GPCRs not only function at the plasma membrane but also at various cellular organelles including endosomes, mitochondria, and Golgi [89]. Although D_1_-related studies are sparse, it will not be a surprise that future studies show D_1_ functional selectivity in different organelles. Location bias is an emerging paradigm in GPCR biology and drug discovery. This is particularly true for endosomes regarding D_1_Rs. D_1_Rs start to internalize through endosomes where β-arrestin is recruited. It has been shown that endocytosis promotes rapid D_1_ signaling [90]. This could be in line with the initial signal at the plasma membrane, but there are studies showing GPCR signaling via heterotrimeric G proteins from endosomes [91]. Therefore, it will not be unexpected that D_1_ has signaling bias toward β-arrestin at endosomes, but more studies are needed for this topic.

## 9. Functionally Selective D_1_ Ligands: Pharmacological Retrospect

**Figure 2:**
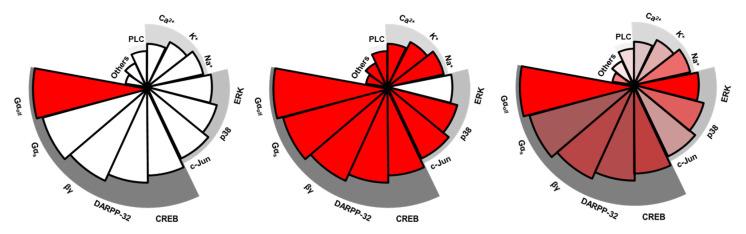
Diagram of functional selectivity. Complete functional selectivity, at the extreme, is a ligand acting as both an agonist and an antagonist at different functions at a single receptor. The left panel exemplifies complete bias with a ligand functioning as an agonist (red color) only at Gα_olf_-mediated cAMP activation. The middle panel exemplifies another complete bias with a ligand functioning as an antagonist (blank) only at β-arrestin-related ERK activation. Incomplete/subtle functional selectivity more commonly is seen in many dopamine ligands. The right panel exemplifies mild bias since all signaling pathways are activated but to different degrees. Each pie represents a signaling pathway; red color indicates pathways are activated but different transparencies reflect they are activated to different levels (e.g., full vs. partial). Note the signaling pathways, such as Gα_olf_-mediated cAMP activation or β-arrestin related ERK activation, are only examples.

Functional selectivity is currently a hot topic in the drug discovery field. Recently, several novel, functionally selective D_1_ ligands were reported [92,93]. They all were biased toward cAMP compared to β-arrestin signaling. Functional selectivity, at the extreme, has a ligand act as both an agonist and antagonist in different cellular functions at a single receptor [1]. An example would be functioning as an agonist at cAMP and an antagonist at β-arrestin (Figure 2). For many dopamine ligands, however, it is more common to activate all signaling pathways but to different degrees. This incomplete/subtle functional selectivity was first reported by our group using a series of full and partial D_1_ agonists [71]. Full and partial agonists were defined by activation of the canonical D_1_ signaling pathway—the intrinsic activity at stimulating adenylate cyclase and producing cAMP. It is noteworthy that a retrospective review of some “traditional/classical” D_1_ ligands may have revealed that they are functionally selective even if this was not appreciated at the time of publication, as highlighted by our group [71].

Lewis et al. compared the intrinsic activities at adenylate cyclase for a series of structurally dissimilar full and partial D_1_ agonists and their ability to cause functional desensitization [94]. Surprisingly at the time, they found a dissociation between these two activities. For example, three full D_1_ agonists (dihydrexidine, SKF82958, A77636) caused homologous desensitization of the D_1_R in vitro to the same extent as dopamine, whereas two other full agonists (dinapsoline, A68930) and all the partial agonists (SKF38393, pergolide, LSD) caused only partial desensitization that was homologous, but not associated with PKA-induced phosphorylation. Ryman-Rasmussen et al. later tested 13 D_1_ agonists and showed that internalization efficacy was independent of either agonist affinity or chemotype [95]. For example, four agonists from two chemotypes were able to activate adenylate cyclase fully without inducing internalization. A follow-up study further confirmed that ligand-specific differential effects on receptor recycling involved aspects of D_1_Rs that are distal from the ligand-binding domain [96]. Since receptor recycling (i.e., desensitization, internalization) is initiated by β-arrestin recruitment, it is logical to conclude that a retrospective review of these data actually provides proof that these compounds have some degree of functional selectivity between canonical cAMP signaling and novel β-arrestin-related signaling. Moreover, these data are consistent with the hypothesis that functional selectivity reflects subtle ligand-induced conformational changes, as opposed to simple agonist trafficking among discrete receptor active states.

## 10. Prospect on Future Studies of Function Selectivity

Functional selectivity was first reported as a pharmacological phenomenon. Later studies using behavioral and neurophysiological methods, and recent clinical trials, have led to improved understanding of functionally selective drugs and provided significant insight on how to further investigate this “so called” pharmacological phenomenon. The earliest example of functional selectivity was for a series of D_2_ ligands that were full agonists at adenylate cyclase but antagonists at other functions [97,98,99]. The translated in vivo behavioral effects of these highly functionally selective compounds indicated they were uncharacteristic of typical D_2_ agonists [100,101]. Contemporaneous studies of OPC-14597 (later named aripiprazole) showed that its unusual properties most likely involved functional selectivity at the D_2_Rs and possibly 5-HT_1A_ receptors [1,102,103], contrary to later views of it functioning as a simple partial agonist. Spurred by studies emerging at the same time in serotonin [104], angiotensin [17], and opioid [105] systems, both basic research and drug discovery have exploded in the past decade with numerous exciting findings on functional selectivity. One timely area relates to the opioid epidemic in which the search for functionally selective opioid receptor ligands indicated superior analgesic action with decreased addictive or other unwanted properties [106,107,108,109]. Recently, our group reported a landmark neurophysiological study on the functional selectivity of D_1_R-mediated cAMP and β-arrestin signaling. Using a pair of D_1_ agonists with distinct signaling profiles, we evaluated rodent behavior in a T-maze task and examined how this was associated with neural activities in the prefrontal cortex [71]. We showed significant neurophysiological changes correlated with the level of β-arrestin recruitment. These results indicated the feasibility of using neurophysiological measurements as markers for studying D_1_R functional selectivity. It is encouraging that the field has discovered more ways to investigate functional selectivity—not only by pharmacological means but also behavioral and neurophysiological methods. These interdisciplinary approaches improve the innovation and development of more functionally selective D_1_ ligands as better therapeutics.

## 11. Summary

The understanding of functional selectivity has evolved over the years in conjunction with advances in improved knowledge of fundamental receptor signaling and complexes. With the discovery of many novel signaling or sub-pathways related to D_1_Rs, studies related to grasping the breadth of D_1_R functional selectivity are expanding. Even though some reports at the time of publishing did not focus on functional selectivity, a retrospective review of their findings indicate they contributed to this field. More importantly, many studies showed positive implications for each unique D_1_ signaling pathway, suggesting that functional selectivity could be a promising strategy for drug discovery. Furthermore, retrospective pharmacological review revealed that many D_1_ ligands have some degree of mild functional selectivity. Moreover, novel compounds with extreme bias at D_1_ signaling were reported recently. Collectively, these data show that the development of precision medicine with the use of functionally selective D_1_ ligands is a promising direction to pursue.

## References

[1] Urban J., Clarke W., Von Zastrow M., Nichols D.E., Kobilka B., Weinstein H., Javitch J., Roth B.L., Christopoulos A., Sexton P. (2006). Functional Selectivity and Classical Concepts of Quantitative Pharmacology. J. Pharmacol. Exp. Ther..

[2] Kebabian J.W., Greengard P. (1971). Dopamine-Sensitive Adenyl Cyclase: Possible Role in Synaptic Transmission. Science.

[3] Lorenzen E., Ceraudo E., Berchiche Y.A., Rico C.A., Fürstenberg A., Sakmar T.P., Huber T. (2018). G protein subtype–specific signaling bias in a series of CCR5 chemokine analogs. Sci. Signal..

[4] Neve K.A., Seamans J.K., Trantham-Davidson H. (2004). Dopamine receptor signaling. J. Recept. Signal Transduct. Res..

[5] Lefkowitz R.J., Shenoy S.K. (2005). Transduction of receptor signals by beta-arrestins. Science.

[6] Santini E., Feyder M., Gangarossa G., Bateup H.S., Greengard P., Fisone G. (2012). Dopamine- and cAMP-regulated phosphoprotein of 32-kDa (DARPP-32)-dependent activation of extracellular signal-regulated kinase (ERK) and mammalian target of rapamycin complex 1 (mTORC1) signaling in experimental parkinsonism. J. Biol. Chem..

[7] De Rooij J., Zwartkruis F.J.T., Verheijen M.H.G., Cool R.H., Nijman S.M.B., Wittinghofer A., Bos J.L. (1998). Epac is a Rap1 guanine-nucleotide-exchange factor directly activated by cyclic AMP. Nature.

[8] Gamo N.J., Lur G., Higley M.J., Wang M., Paspalas C.D., Vijayraghavan S., Yang Y., Ramos B.P., Peng K., Kata A. (2015). Stress Impairs Prefrontal Cortical Function via D1 Dopamine Receptor Interactions With Hyperpolarization-Activated Cyclic Nucleotide-Gated Channels. Biol. Psychiatry.

[9] Lee S.-M., Yang Y., Mailman R.B. (2014). Dopamine D1 receptor signaling: Does GαQ-phospholipase C actually play a role?. J. Pharmacol. Exp. Ther..

[10] Zhuang X., Belluscio L., Hen R. (2000). GOLFalpha Mediates Dopamine D1 Receptor Signaling. J. Neurosci..

[11] Corvol J.C., Studler J.M., Schonn J.S., Girault J.A., Herve D. (2001). Galpha(olf) is necessary for coupling D1 and A2a receptors to adenylyl cyclase in the striatum. J. Neurochem..

[12] Herve D., Levi-Strauss M., Marey-Semper I., Verney C., Tassin J.P., Glowinski J., Girault J.A. (1993). G olf and G s in rat basal ganglia: Possible involvement of G olf in the coupling of dopamine D 1 receptor with adenylyl cyclase. J. Neurosci..

[13] Watson J.B., Coulter P.M., Margulies J.E., de Lecea L., Danielson P.E., Erlander M.G., Sutcliffe J.G. (1994). G-protein gamma 7 subunit is selectively expressed in medium-sized neurons and dendrites of the rat neostriatum. J. Neurosci. Res..

[14] Wang Q., Jolly J.P., Surmeier J.D., Mullah B.M., Lidow M.S., Bergson C.M., Robishaw J.D. (2001). Differential dependence of the D1 and D5 dopamine receptors on the G protein gamma 7 subunit for activation of adenylylcyclase. J. Biol. Chem..

[15] Lovell K.M., Frankowski K.J., Stahl E.L., Slauson S.R., Yoo E., Prisinzano T.E., Aube J., Bohn L.M. (2015). Structure-activity relationship studies of functionally selective kappa opioid receptor agonists that modulate ERK 1/2 phosphorylation while preserving G protein over betaarrestin2 signaling bias. ACS Chem. Neurosci..

[16] Khajehali E., Malone D.T., Glass M., Sexton P., Christopoulos A., Leach K. (2015). Biased Agonism and Biased Allosteric Modulation at the CB1 Cannabinoid Receptor. Mol. Pharmacol..

[17] Turu G., Balla A., Hunyady L. (2019). The Role of β-Arrestin Proteins in Organization of Signaling and Regulation of the AT1 Angiotensin Receptor. Front. Endocrinol..

[18] Aringhieri S., Kolachalam S., Gerace C., Carli M., Verdesca V., Brunacci M.G., Rossi C., Ippolito C., Solini A., Corsini G.U. (2017). Clozapine as the most efficacious antipsychotic for activating ERK 1/2 kinases: Role of 5-HT2A receptor agonism. Eur. Neuropsychopharm..

[19] Brame A.L., Maguire J.J., Yang P., Dyson A., Torella R., Cheriyan J., Singer M., Glen R.C., Wilkinson I.B., Davenport A.P. (2015). Design, Characterization, and First-In-Human Study of the Vascular Actions of a Novel Biased Apelin Receptor Agonist. Hypertension.

[20] Chebani Y., Marion C., Zizzari P., Chettab K., Pastor M., Korostelev M., Geny D., Epelbaum J., Tolle V., Morisset-Lopez S. (2016). Enhanced responsiveness of Ghsr Q343X rats to ghrelin results in enhanced adiposity without increased appetite. Sci. Signal..

[21] Sykes D., Riddy D., Stamp C., Bradley M.E., McGuiness N., Sattikar A., Guerini D., Rodrigues I., Glaenzel A., Dowling M.R. (2014). Investigating the molecular mechanisms through which FTY720-P causes persistent S1P1receptor internalization. Br. J. Pharmacol..

[22] Möller D., Kling R.C., Skultety M., Leuner K., Hübner H., Gmeiner P. (2014). Functionally Selective Dopamine D2, D3 Receptor Partial Agonists. J. Med. Chem..

[23] Rose S.J., Pack T.F., Peterson S.M., Payne K., Borrelli E., Caron M.G. (2018). Engineered D2R Variants Reveal the Balanced and Biased Contributions of G-Protein and beta-Arrestin to Dopamine-Dependent Functions. Neuropsychopharmacology.

[24] Chen J., Rusnak M., Luedtke R.R., Sidhu A. (2004). D1 Dopamine Receptor Mediates Dopamine-induced Cytotoxicity via the ERK Signal Cascade. J. Biol. Chem..

[25] Urs N.M., Daigle T.L., Caron M.G. (2011). A dopamine D1 receptor-dependent beta-arrestin signaling complex potentially regulates morphine-induced psychomotor activation but not reward in mice. Neuropsychopharmacology.

[26] Urs N.M., Bido S., Peterson S.M., Daigle T.L., Bass C.E., Gainetdinov R.R., Bezard E., Caron M.G. (2015). Targeting beta-arrestin2 in the treatment of L-DOPA-induced dyskinesia in Parkinson’s disease. Proc. Natl. Acad. Sci. USA.

[27] Brami-Cherrier K., Valjent E., Garcia M., Pagès C., Hipskind R.A., Caboche J. (2002). Dopamine Induces a PI3-Kinase-Independent Activation of Akt in Striatal Neurons: A New Route to cAMP Response Element-Binding Protein Phosphorylation. J. Neurosci..

[28] Nagai T., Takuma K., Kamei H., Ito Y., Nakamichi N., Ibi D., Nakanishi Y., Murai M., Mizoguchi H., Nabeshima T. (2007). Dopamine D1 receptors regulate protein synthesis-dependent long-term recognition memory via extracellular signal-regulated kinase 1/2 in the prefrontal cortex. Learn. Mem..

[29] Zhen X., Uryu K., Wang H.-Y., Friedman E. (1998). D1Dopamine Receptor Agonists Mediate Activation of p38 Mitogen-Activated Protein Kinase and c-Jun Amino-Terminal Kinase by a Protein Kinase A-Dependent Mechanism in SK-N-MC Human Neuroblastoma Cells. Mol. Pharmacol..

[30] Santini E., Valjent E., Usiello A., Carta M., Borgkvist A., Girault J.-A., Herve D., Greengard P., Fisone G. (2007). Critical Involvement of cAMP/DARPP-32 and Extracellular Signal-Regulated Protein Kinase Signaling in L-DOPA-Induced Dyskinesia. J. Neurosci..

[31] Weissman J.T., Ma J.-N., Essex A., Gao Y., Burstein E.S. (2004). G-protein-coupled receptor-mediated activation of rap GTPases: Characterization of a novel Gαi regulated pathway. Oncogene.

[32] Cull-Candy S., Kelly L., Farrant M. (2006). Regulation of Ca^2+^-permeable AMPA receptors: Synaptic plasticity and beyond. Curr. Opin. Neurobiol..

[33] Surmeier D., Bargas J., Hemmings H.C., Nairn A.C., Greengard P. (1995). Modulation of calcium currents by a D1 dopaminergic protein kinase/phosphatase cascade in rat neostriatal neurons. Neuron.

[34] Hernández-López S., Bargas J., Surmeier D.J., Reyes A., Galarraga E. (1997). D1Receptor Activation Enhances Evoked Discharge in Neostriatal Medium Spiny Neurons by Modulating an L-Type Ca^2+^ Conductance. J. Neurosci..

[35] Takahashi T., Momiyama A. (1993). Different types of calcium channels mediate central synaptic transmission. Nat. Cell Biol..

[36] Kisilevsky A.E., Mulligan S.J., Altier C., Iftinca M.C., Varela D., Tai C., Chen L., Hameed S., Hamid J., MacVicar B.A. (2008). D1 Receptors Physically Interact with N-Type Calcium Channels to Regulate Channel Distribution and Dendritic Calcium Entry. Neuron.

[37] Hopf F.W., Cascini M.G., Gordon A.S., Diamond I., Bonci A. (2003). Cooperative activation of dopamine D1 and D2 receptors increases spike firing of nucleus accumbens neurons via G-protein betagamma subunits. J. Neurosci..

[38] Gorelova N., Seamans J.K., Yang C.R. (2002). Mechanisms of Dopamine Activation of Fast-Spiking Interneurons That Exert Inhibition in Rat Prefrontal Cortex. J. Neurophysiol..

[39] Yang C.R., Seamans J.K. (1996). Dopamine D1 receptor actions in layers V-VI rat prefrontal cortex neurons in vitro: Modulation of dendritic-somatic signal integration. J. Neurosci..

[40] Dong Y., White F.J. (2003). Dopamine D1-Class Receptors Selectively Modulate a Slowly Inactivating Potassium Current in Rat Medial Prefrontal Cortex Pyramidal Neurons. J. Neurosci..

[41] Dong Y., Cooper D., Nasif F., Hu X.-T., White F.J. (2004). Dopamine Modulates Inwardly Rectifying Potassium Currents in Medial Prefrontal Cortex Pyramidal Neurons. J. Neurosci..

[42] Calabresi P., Mercuri N., Stanzione P., Stefani A., Bernardi G. (1987). Intracellular studies on the dopamine-induced firing inhibition of neostriatal neurons in vitro: Evidence for D1 receptor involvement. Neuroscience.

[43] Surmeier D.J., Eberwine J., Wilson C.J., Cao Y., Stefani A., Kitai S.T. (1992). Dopamine receptor subtypes colocalize in rat striatonigral neurons. Proc. Natl. Acad. Sci. USA.

[44] Schiffmann S., Lledo P.M., Vincent J.D. (1995). Dopamine D1 receptor modulates the voltage-gated sodium current in rat striatal neurones through a protein kinase A. J. Physiol..

[45] Schiffmann S.N., Desdouits F., Menu R., Greengard P., Vincent J.D., Vanderhaeghen J.J., Girault J.A. (1998). Modulation of the voltage-gated sodium current in rat striatal neurons by DARPP-32, an inhibitor of protein phosphatase. Eur. J. Neurosci..

[46] Cantrell A.R., Smith R.D., Goldin A.L., Scheuer T., Catterall W.A. (1997). Dopaminergic modulation of sodium current in hippocampal neurons via cAMP-dependent phosphorylation of specific sites in the sodium channel alpha subunit. J. Neurosci..

[47] Iwamoto T., Okumura S., Iwatsubo K., Kawabe J.-I., Ohtsu K., Sakai I., Hashimoto Y., Izumitani A., Sango K., Ajiki K. (2003). Motor Dysfunction in Type 5 Adenylyl Cyclase-null Mice. J. Biol. Chem..

[48] Lee K.-W., Hong J.-H., Choi I.Y., Che Y., Lee J.-K., Yang S.-D., Song C.-W., Kang H.S., Lee J.-H., Noh J.S. (2002). Impaired D2 Dopamine Receptor Function in Mice Lacking Type 5 Adenylyl Cyclase. J. Neurosci..

[49] Wang H.Y., Undie A.S., Friedman E. (1995). Evidence for the coupling of Gq protein to D1-like dopamine sites in rat striatum: Possible role in dopamine-mediated inositol phosphate formation. Mol. Pharmacol..

[50] Mahan L.C., Burch R.M., Monsma F.J., Sibley D.R. (1990). Expression of striatal D1 dopamine receptors coupled to inositol phosphate production and Ca^2+^ mobilization in Xenopus oocytes. Proc. Natl. Acad. Sci. USA.

[51] Undie A.S., Friedman E. (1990). Stimulation of a dopamine D1 receptor enhances inositol phosphates formation in rat brain. J. Pharmacol. Exp. Ther..

[52] Undie A.S., Weinstock J., Sarau H.M., Friedman E. (2008). Evidence for a Distinct D1Like Dopamine Receptor that Couples to Activation of Phosphoinositide Metabolism in Brain. J. Neurochem..

[53] Jin L.-Q., Goswami S., Cai G., Zhen X., Friedman E. (2003). SKF83959 selectively regulates phosphatidylinositol-linked D1 dopamine receptors in rat brain. J. Neurochem..

[54] Zhen X., Goswami S., Friedman E. (2005). The role of the phosphatidyinositol-linked D1 dopamine receptor in the pharmacology of SKF83959. Pharmacol. Biochem. Behav..

[55] Lee S.-M., Kant A., Blake D., Murthy V., Boyd K., Wyrick S.J., Mailman R.B. (2014). SKF-83959 is not a highly-biased functionally selective D1 dopamine receptor ligand with activity at phospholipase C. Neuropharmacology.

[56] Chun L.S., Free R.B., Doyle T.B., Huang X.-P., Rankin M.L., Sibley D.R. (2013). D1-D2Dopamine Receptor Synergy Promotes Calcium Signaling via Multiple Mechanisms. Mol. Pharmacol..

[57] Haack K.K., Mccarty N.A. (2011). Functional Consequences of GPCR Heterodimerization: GPCRs as Allosteric Modulators. Pharmaceuticals.

[58] Prinster S.C., Hague C., Hall R.A. (2005). Heterodimerization of G Protein-Coupled Receptors: Specificity and Functional Significance. Pharmacol. Rev..

[59] Hübner H., Schellhorn T., Gienger M., Schaab C., Kaindl J., Leeb L., Clark T., Möller D., Gmeiner P. (2016). Structure-guided development of heterodimer-selective GPCR ligands. Nat. Commun..

[60] Espinoza S., Salahpour A., Masri B., Sotnikova T.D., Messa M., Barak L.S., Caron M.G., Gainetdinov R.R. (2011). Functional Interaction between Trace Amine-Associated Receptor 1 and Dopamine D2 Receptor. Mol. Pharmacol..

[61] Rashid A.J., O’Dowd B.F., Verma V., George S.R. (2007). Neuronal Gq/11-coupled dopamine receptors: An uncharted role for dopamine. Trends Pharmacol. Sci..

[62] Hasbi A., Fan T., Alijaniaram M., Nguyen T., Perreault M., O’Dowd B.F., George S.R. (2009). Calcium signaling cascade links dopamine D1–D2 receptor heteromer to striatal BDNF production and neuronal growth. Proc. Natl. Acad. Sci. USA.

[63] Rashid A.J., So C.H., Kong M.M.C., Furtak T., El-Ghundi M., Cheng R., O’Dowd B.F., George S.R. (2007). D1-D2 dopamine receptor heterooligomers with unique pharmacology are coupled to rapid activation of Gq/11 in the striatum. Proc. Natl. Acad. Sci. USA.

[64] Hasbi A., O’Dowd B.F., George S.R. (2011). Dopamine D1-D2 receptor heteromer signaling pathway in the brain: Emerging physiological relevance. Mol. Brain.

[65] Bateup H.S., Svenningsson P., Kuroiwa M., Gong S., Nishi A., Heintz N., Greengard P. (2008). Cell type–specific regulation of DARPP-32 phosphorylation by psychostimulant and antipsychotic drugs. Nat. Neurosci..

[66] Tritsch N.X., Sabatini B.L. (2012). Dopaminergic Modulation of Synaptic Transmission in Cortex and Striatum. Neuron.

[67] Glatt C.E., Snyder S.H. (1993). Cloning and expression of an adenylyl cyclase localized to the corpus striatum. Nat. Cell Biol..

[68] Labonte B., Engmann O., Purushothaman I., Menard C., Wang J., Tan C., Scarpa J.R., Moy G., Loh Y.E., Cahill M. (2017). Sex-specific transcriptional signatures in human depression. Nat. Med..

[69] Beaulieu J.M., Sotnikova T.D., Marion S., Lefkowitz R.J., Gainetdinov R.R., Caron M.G. (2005). An Akt/beta-arrestin 2/PP2A signaling complex mediates dopaminergic neurotransmission and behavior. Cell.

[70] Liu X., Ma L., Li H.H., Huang B., Li Y.X., Tao Y.Z., Ma L. (2015). Beta-Arrestin-biased signaling mediates memory reconsolidation. Proc. Natl. Acad. Sci. USA.

[71] Yang Y., Lee S., Imamura F., Gowda K., Amin S., Mailman R.B. (2021). D1 dopamine receptors intrinsic activity and functional selectivity affect working memory in prefrontal cortex. Mol. Psychiatry.

[72] Klyubin I., Betts V., Welzel A.T., Blennow K., Zetterberg H., Wallin A., Lemere C.A., Cullen W.K., Peng Y., Wisniewski T. (2008). Amyloid beta protein dimer-containing human CSF disrupts synaptic plasticity: Prevention by systemic passive immunization. J. Neurosci..

[73] Thathiah A., Horre K., Snellinx A., Vandewyer E., Huang Y., Ciesielska M., De Kloe G., Munck S., De Strooper B. (2013). Beta-arrestin 2 regulates Abeta generation and gamma-secretase activity in Alzheimer’s disease. Nat. Med..

[74] Jiang T., Yu J.T., Tan M.S., Zhu X.C., Tan L. (2013). beta-Arrestins as potential therapeutic targets for Alzheimer’s disease. Mol. Neurobiol..

[75] Huang X., Lewis M.M., Van Scoy L.J., De Jesus S., Eslinger P.J., Arnold A.C., Miller A.J., Fernandez-Mendoza J., Snyder B., Harrington W. (2020). The D1/D5 Dopamine Partial Agonist PF-06412562 in Advanced-Stage Parkinson’s Disease: A Feasibility Study. J. Park. Dis..

[76] Riesenberg R., Werth J., Zhang Y., Duvvuri S., Gray D. (2020). PF-06649751 efficacy and safety in early Parkinson’s disease: A randomized, placebo-controlled trial. Ther. Adv. Neurol. Disord..

[77] Papapetropoulos S., Liu W., Duvvuri S., Thayer K., Gray D.L. (2018). Evaluation of D1/D5 Partial Agonist PF-06412562 in Parkinson’s Disease following Oral Administration. Neurodegener. Dis..

[78] Wang M., Datta D., Enwright J., Galvin V., Yang S.-T., Paspalas C., Kozak R., Gray D.L., Lewis D.A., Arnsten A.F. (2019). A novel dopamine D1 receptor agonist excites delay-dependent working memory-related neuronal firing in primate dorsolateral prefrontal cortex. Neuropharmacology.

[79] Balice-Gordon R., Honey G.D., Chatham C., Arce E., Duvvuri S., Naylor M.G., Liu W., Xie Z., DeMartinis N., Harel B.T. (2020). A Neurofunctional Domains Approach to Evaluate D1/D5 Dopamine Receptor Partial Agonism on Cognition and Motivation in Healthy Volunteers with Low Working Memory Capacity. Int. J. Neuropsychopharmacol..

[80] Arce E., Balice-Gordon R., Duvvuri S., Naylor M., Xie Z., Harel B., Kozak R., Gray D.L., DeMartinis N. (2019). A novel approach to evaluate the pharmacodynamics of a selective dopamine D1/D5 receptor partial agonist (PF-06412562) in patients with stable schizophrenia. J. Psychopharmacol..

[81] Sun B., Feng D., Chu M.L.-H., Fish I., Lovera S., Sands Z.A., Kelm S., Valade A., Wood M., Ceska T. (2021). Crystal structure of dopamine D1 receptor in complex with G protein and a non-catechol agonist. Nat. Commun..

[82] Kalani M.Y., Vaidehi N., Hall S.E., Trabanino R.J., Freddolino P.L., Floriano W., Kam V.W.T., Goddard W.A. (2004). The predicted 3D structure of the human D2 dopamine receptor and the binding site and binding affinities for agonists and antagonists. Proc. Natl. Acad. Sci. USA.

[83] Chien E.Y.T., Liu W., Cherezov V., Stevens R.C., Zhao Q., Katritch V., Han G.W., Hanson M.A., Shi L., Newman A.H. (2010). Structure of the Human Dopamine D3 Receptor in Complex with a D2/D3 Selective Antagonist. Science.

[84] Wang S., Wacker D., Levit A., Che T., Betz R.M., McCorvy J.D., Venkatakrishnan A.J., Huang X.-P., Dror R.O., Shoichet B.K. (2017). D4dopamine receptor high-resolution structures enable the discovery of selective agonists. Science.

[85] Yin J., Chen K.-Y.M., Clark M.J., Hijazi M., Kumari P., Bai X.-C., Sunahara R.K., Barth P., Rosenbaum D.M. (2020). Structure of a D2 dopamine receptor–G-protein complex in a lipid membrane. Nat. Cell Biol..

[86] Fowler J.C., Bhattacharya S., Urban J.D., Vaidehi N., Mailman R.B. (2012). Receptor Conformations Involved in Dopamine D2L Receptor Functional Selectivity Induced by Selected Transmembrane-5 Serine Mutations. Mol. Pharmacol..

[87] Zhang H., Unal H., Desnoyer R., Han G.W., Patel N., Katritch V., Karnik S.S., Cherezov V., Stevens R.C. (2015). Structural Basis for Ligand Recognition and Functional Selectivity at Angiotensin Receptor. J. Biol. Chem..

[88] Aslanoglou D., Bertera S., Sánchez-Soto M., Free R.B., Lee J., Zong W., Xue X., Shrestha S., Brissova M., Logan R.W. (2021). Dopamine regulates pancreatic glucagon and insulin secretion via adrenergic and dopaminergic receptors. Transl. Psychiatry.

[89] Nezhady M.A.M., Rivera J.C., Chemtob S. (2020). Location Bias as Emerging Paradigm in GPCR Biology and Drug Discovery. iScience.

[90] Kotowski S.J., Hopf F.W., Seif T., Bonci A., von Zastrow M. (2011). Endocytosis Promotes Rapid Dopaminergic Signaling. Neuron.

[91] Tsvetanova N.G., Irannejad R., von Zastrow M. (2015). G Protein-coupled Receptor (GPCR) Signaling via Heterotrimeric G Proteins from Endosomes. J. Biol. Chem..

[92] Gray D.L., Allen J.A., Mente S., O’Connor R.E., DeMarco G.J., Efremov I., Tierney P., Volfson D., Davoren J., Guilmette E. (2018). Impaired beta-arrestin recruitment and reduced desensitization by non-catechol agonists of the D1 dopamine receptor. Nat. Commun..

[93] Kozak R., Kiss T., Dlugolenski K., Johnson D.E., Gorczyca R.R., Kuszpit K., Harvey B.D., Stolyar P., Rizzo S.J.S., Hoffmann W.E. (2020). Characterization of PF-6142, a Novel, Non-Catecholamine Dopamine Receptor D1 Agonist, in Murine and Nonhuman Primate Models of Dopaminergic Activation. Front. Pharmacol..

[94] Lewis M.M., Watts V.J., Lawler C.P., Nichols D.E., Mailman R. (1998). Homologous desensitization of the D1A dopamine receptor: Efficacy in causing desensitization dissociates from both receptor occupancy and functional potency. J. Pharmacol. Exp. Ther..

[95] Ryman-Rasmussen J.P., Nichols D.E., Mailman R.B. (2005). Differential Activation of Adenylate Cyclase and Receptor Internalization by Novel Dopamine D1 Receptor Agonists. Mol. Pharmacol..

[96] Ryman-Rasmussen J.P., Griffith A., Oloff S., Vaidehi N., Brown J.T., Goddard W.A., Mailman R.B. (2007). Functional selectivity of dopamine D1 receptor agonists in regulating the fate of internalized receptors. Neuropharmacology.

[97] Mottola D.M., Cook L.L., Jones S.R., Booth R.G., Nichols D.E., Mailman R.B. (1991). Dihydrexidine, a selective dopamine receptor agonist that may discriminate postsynaptic D 2 receptors. Soc. Neurosci. Abstr..

[98] Mottola D.M., Kilts J.D., Lewis M.M., Connery H.S., Walker Q.D., Jones S., Booth R.G., Hyslop D.K., Piercey M., Wightman R.M. (2002). Functional Selectivity of Dopamine Receptor Agonists. I. Selective Activation of Postsynaptic Dopamine D2Receptors Linked to Adenylate Cyclase. J. Pharmacol. Exp. Ther..

[99] Kilts J.D., Connery H.S., Arrington E.G., Lewis M.M., Lawler C.P., Oxford G.S., O’Malley K.L., Todd R.D., Blake B.L., Nichols D.E. (2002). Functional Selectivity of Dopamine Receptor Agonists. II. Actions of Dihydrexidine in D2LReceptor-Transfected MN9D Cells and Pituitary Lactotrophs. J. Pharmacol. Exp. Ther..

[100] Darney K.J., Lewis M.H., Brewster W.K., Nichols D.E., Mailman R. (1991). Behavioral effects in the rat of dihydrexidine, a high-potency, full-efficacy D1 dopamine receptor agonist. Neuropsychopharmacology.

[101] Smith H.P., Nichols D.E., Mailman R.B., Lawler C.P. (1997). Locomotor inhibition, yawning and vacuous chewing induced by a novel dopamine D2 post-synaptic receptor agonist. Eur. J. Pharmacol..

[102] Shapiro D.A., Renock S., Arrington E., Chiodo L.A., Liu L.-X., Sibley D.R., Roth B.L., Mailman R. (2003). Aripiprazole, A Novel Atypical Antipsychotic Drug with a Unique and Robust Pharmacology. Neuropsychopharmacology.

[103] Urban J., Vargas G.A., Von Zastrow M., Mailman R.B. (2006). Aripiprazole has Functionally Selective Actions at Dopamine D2 Receptor-Mediated Signaling Pathways. Neuropsychopharmacology.

[104] Berg K.A., Clarke W.P., Maayani S., Goldfarb J. (1998). Pleiotropic behavior of 5-HT2A and 5-HT2C receptor agonists. Ann. N. Y. Acad. Sci..

[105] Whistler J.L., Chuang H.H., Chu P., Jan L.Y., von Zastrow M. (1999). Functional dissociation of mu opioid receptor signaling and endocytosis: Implications for the biology of opiate tolerance and addiction. Neuron.

[106] Manglik A., Lin H., Aryal D.K., McCorvy J.D., Dengler D., Corder G., Levit A., Kling R.C., Bernat V., Hübner H. (2016). Structure-based discovery of opioid analgesics with reduced side effects. Nature.

[107] Viscusi E.R., Webster L.R., Kuss M.E., Daniels S.E., Bolognese J.A., Zuckerman S., Soergel D.G., Subach R.A., Cook E., Skobieranda F. (2016). A randomized, phase 2 study investigating TRV130, a biased ligand of the μ-opioid receptor, for the intravenous treatment of acute pain. Pain.

[108] White K.L., Robinson J.E., Zhu H., DiBerto J.F., Polepally P.R., Zjawiony J.K., Nichols D.E., Malanga C.J., Roth B.L. (2015). The G protein-biased kappa-opioid receptor agonist RB-64 is analgesic with a unique spectrum of activities in vivo. J. Pharmacol. Exp. Ther..

[109] White K.L., Scopton A.P., Rives M.L., Bikbulatov R.V., Polepally P.R., Brown P.J., Kenakin T., Javitch J.A., Zjawiony J.K., Roth B.L. (2014). Identification of novel functionally selective kappa-opioid receptor scaffolds. Mol. Pharmacol..

